# A retrospective comparison of two protocols for correction of skeletal Class III malocclusion in prepubertal children: hybrid hyrax expander with mandibular miniplates and rapid maxillary expansion with face mask

**DOI:** 10.1186/s40510-022-00446-z

**Published:** 2023-01-23

**Authors:** Nour Eldin Tarraf, Oyku Dalci, Kerem Dalci, Ayse Tuba Altug, M. Ali Darendeliler

**Affiliations:** 1 Discipline of Orthodontics and Paediatric Dentistry, Sydney Dental School, Faculty of Medicine and Health, University of Sydney, Sydney, Australia; 2 Department of Orthodontics, Faculty of Dentistry, Ankara University, Ankara, Turkey

## Abstract

**Background:**

This study compared the skeletal and dental effects of a hybrid maxillary expander with mandibular miniplates (HE-MP) and Class III elastics to conventional tooth-borne rapid maxillary expander and face mask (RME–FM) in skeletal Class III treatment.

**Methods:**

This retrospective study included 36 skeletal Class III patients. Eighteen patients (mean age 10.24 ± 1.31 years) were treated with a hybrid expander, two mandibular L-shaped miniplates and full-time Class III elastics (HE-MP group). Their results were compared to a group of patients treated with conventional RME–FM (*n* = 18; mean age 10.56 ± 1.41 year). Radiographs were taken before (T1) and after treatment (T2). All patients were in cervical maturation stages CS1–CS3 at T1. The measured outcomes were the changes in sagittal and vertical skeletal and dental cephalometric measurements.

**Results:**

Treatment time was approximately 15.5 ± 2.8 months with the HE-MP and 11.85 ± 3.41 months for the RME–FM. The Class III malocclusion was corrected in both groups with significant changes. The maxilla advanced more in the HE-MP group, with an increase in SNA of 4.26° ± 2.15° compared to 1.14 ± 0.93 in the RME–FM group *(p* < 0.001). The effect on the mandible was similar in both groups, while the overall skeletal change was significantly greater with HE-MP, with an increase in the ANB of 5.25° ± 2.03° and a Wits appraisal increase of 6.03 ± 3.13 mm, as opposed to 2.04° ± 1.07° and 2.94 ± 1.75 mm with the RME–FM (*p* < 0.001). Dental changes were significantly higher with RME–FM, with an increase in incisor inclination (U1-SN) of 5.02° ± 3.93° (*p* < 0.001), with no significant changes in the HE-MP group. The mandibular incisors retroclined by 5.29° ± 3.57° at L1-MP with the RME–FM, while they advanced slightly with the HE-MP by 2.87° ± 5.37° (*p* < 0.001).

**Conclusion:**

The use of skeletal anchorage for maxillary expansion and protraction significantly increases skeletal effects and reduces dental side effects compared to tooth-borne maxillary expansion and protraction. These results need to be investigated in the long term.

## Introduction

Class III malocclusion can result from maxillary deficiency, mandibular prognathism or a combination of both [1]. In growing children, the most commonly used treatment approach is the protraction face mask that aims to stimulate maxillary growth in a downward and forward direction and redirect mandibular growth [2]. Rapid maxillary expansion (RME) is commonly combined with face mask (FM) therapy, as it is thought that the expansion may facilitate sutural response to the protraction forces; however, the literature is divided on this point [2, 3]. Nevertheless, there are several limitations to face mask therapy. Firstly, the cumbersome nature of the extraoral appliance limits patient acceptance and compliance, and thus only part-time wear is possible [2]. Secondly, in the conventional use of the RME–FM combination, the appliance is tooth-borne, resulting in dental side effects caused by mesial migration of the maxillary dentition, proclination of the upper incisors with increased anterior crowding, and lingual tipping of the mandibular incisors [2, 4]. Additionally, the technique has the effect of rotating the mandible backward, thus increasing the lower anterior face height, which may be unfavourable, especially in high-angle cases [4].

The introduction of skeletal anchorage using miniplates by De Clerck et al. [5] allowed the use of purely bone-anchored maxillary protraction (BAMP), thus eliminating dental side effects while also allowing the forces to be applied for 24 h a day. This approach was proposed as a method to produce significant skeletal changes in managing maxillary hypoplasia, while avoiding dental side effects [6]. However, this method does not incorporate maxillary expansion, which in many cases is required due to the transverse maxillary deficiency that usually occurs in Class III cases [7]. Additionally, incorporating maxillary expansion, although there are some conflicting reports, may improve the maxillary response to protraction forces [8], with some authors even suggesting that RME alone may displace the maxilla downwards and forwards [9, 10]. Wilmes et al. introduced the Hybrid Hyrax appliance [10], which shares the load of maxillary expansion and protraction between the first molars and two palatal miniscrews. The majority of the load is carried by the miniscrews, thus reducing the dental side effects and maximising the skeletal effect [11]. The Hybrid Hyrax was combined with a mandibular anchorage plate [12] (coined the ‘Mentoplate’), which is placed in the symphysis apical to the incisors to allow Class III elastic traction. This method was shown to be effective in maxillary protraction and the correction of Class III malocclusion [13] with similar results to those produced with the Hybrid Hyrax and face mask combination [14].

A recent meta-analysis and systematic review compared the effects of various BAMP techniques, including those with mini-implant supported appliances and various force application methods, such as class III elastics or face mask. They concluded that none of the included studies of BAMP techniques were superior to traditional face mask in ANB or Wits changes, however, with a warning of significant heterogeneity of the studies and lack of data on SNA angle [15]. To date, only two studies [13, 14] have assessed the effects of miniscrew-supported maxillary expansion combined with the wearing of Class III elastics to mandibular miniplates, and the effects were not directly compared with those of a conventional RME face mask. The mandibular miniplate placement in DeClerk’s BAMP technique [16] requires lower canine eruption, meaning that treatment cannot typically start until around the age of 11 years, Since the Mentoplate is placed apical to the lower incisors and away from the developing canine, it enables treatment to start earlier [12]. However, compared to the BAMP method [12], Mentoplate surgery is more invasive, with the single plate fixed with three to four bone screws apical to the permanent mandibular incisors after reflecting a single large mucoperiosteal flap [12]. L-shaped miniplates can also be used for the purpose of class III force application and can be placed with a less invasive surgery apical to lower incisors in children younger than 11 years. No studies have analysed the use of this type of miniplate application.

Therefore, this study aimed to compare the skeletal and dental effects of a miniscrew-supported hybrid expander combined with the wear of Class III elastics to miniplates in the anterior mandible with conventional tooth-borne RME face mask therapy.

## Methods

Ethics approval (X20-0456 and 2020/ETH02668) was obtained from the human research Ethics Committee of the Sydney Local Health District.

This retrospective study included 36 Class III malocclusion patients treated with either the hybrid expander and mandibular miniplates with Class III elastics protocol (HE-MP) or tooth-borne RME with face mask therapy (RME–FM).

Inclusion criteria: All patients in both groups presented with a skeletal Class III malocclusion, determined clinically by assessment of the extraoral features of retrusive malar base and concave profile, an anterior crossbite or edge-to-edge relationship and a molar Class III relationship. All were prepubertal in terms of skeletal maturity as assessed by the cervical vertebral maturation index [17] (CS1-CS3) before treatment (T1).

The HE-MP was a group of 18 consecutively treated cases from the first author’s (NET’s) practice between 2013 and 2019. The mean age was 10.24 years (SD = 1.31) with 8 girls and 10 boys (Table 1).

**Table 1:** Age and treatment duration in years for both groups

	HE-MP		RME–FM		*p*
	Mean	SD	Mean	SD	
Age at T1	10.24	1.31	10.56	1.41	0.34
Tx duration	15.5	2.80	11.85	3.41	0.001

An age- and gender-matched active control group of 18 consecutively treated Class III cases, 7 girls and 11 boys, mean age 10.56 years (SD = 1.41) (Table 1), treated at Ankara University, Turkey between 2015 and 2018 were used for comparison.

Both groups had records collected before treatment (T1) and after positive overjet was achieved (T2). In one patient due to poorer elastic compliance and due to the nature of the dental changes with bone-anchored appliances [6], although positive overjet was not achieved, significant facial improvement was obtained, and therefore this patient’s results were also included in the analysis.

### Hybrid expander: mandibular miniplate protocol (HE-MP)

**Fig. 1:**
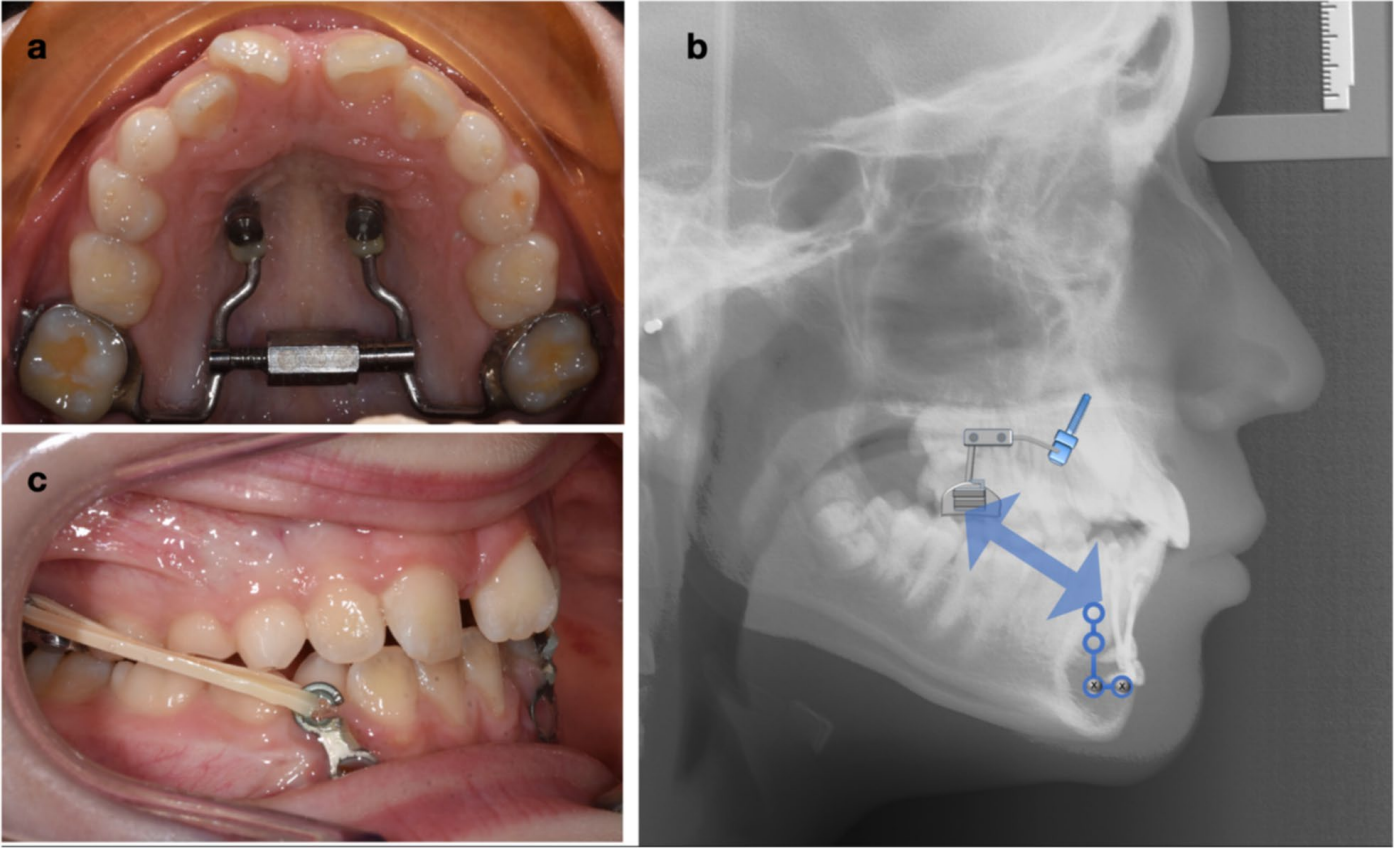
Hybrid expander miniplate (HE-MP) set-up: **A** Hybrid expander with two palatal miniscrews and molar bands with buccal hooks for elastic wear; **B** Schematic representation of the biomechanics and force vector for elastic wear; **C** Elastic band connecting the mandibular miniplate to the hook on the maxillary molar band. The miniplate has been converted into a hook by cutting an opening using a carbide bur

**Fig. 2:**
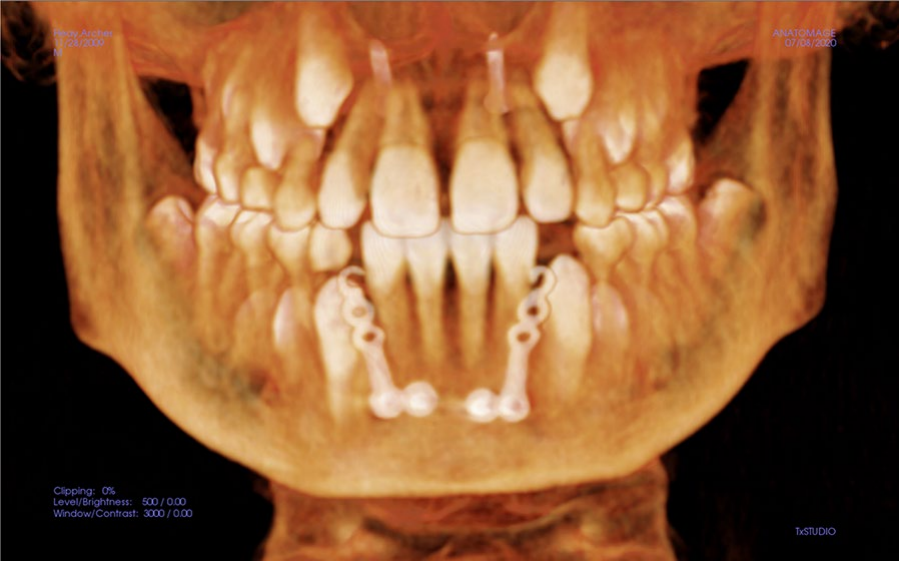
L-plate with two screws placed bilaterally apical to the lower incisors, to avoid the developing mandibular canine

A hybrid expander (Fig. 1a) modified from the Hybrid Hyrax designed by Wilmes et al. [17] was used. Two palatal miniscrews (2 × 9 mm; PSM Medical Solutions, Gunningen, Germany) were placed paramedian on both sides of the midpalatal suture at the third palatine Rugae line, as described by Wilmes et al. [12]. The PowerScrew (Tiger Dental, Bregenz, Austria) was laser-welded to the Benefit abutments (PSM Medical Solutions, Gunningen, Germany). At cementation, the appliance was secured to the miniscrews using two fixation screws (Benefit PSM Medical Solutions, Gunningen, Germany). Patients were instructed to turn the expander once a day (0.17 mm) for 2 weeks ahead of miniplate insertion. Expansion was then continued at a slower rate of 1–2 turns per week until the desired expansion was achieved. Miniplate insertion was performed by an oral surgeon using conventional trauma plates (Stryker Universal Orthognathic; Stryker, Kalamazoo, MI, USA). Two small mucoperiosteal flaps were raised and two L-shaped plates were placed, one on each side (Fig. 1b, c). This was particularly important in the younger patients, whose mandibular canines had not yet erupted. The L-plates were used so that the screws were placed apical to the mandibular central and lateral incisors on each side (Fig. 2). The plate emerged in the attached gingiva or just at the junction of attached and unattached gingiva. After an initial 8-week period of healing, during which the maxillary expansion was being completed, the tops of the plates were converted to hooks using a high-speed carbide bur. Elastics were started with gradually increasing strength, similar to what was recommended by De Clerck et al. [18], in order to gradually increase the bone density around the miniplates and increase their stability [19] (Fig. 1c). For the first 6 weeks, 100 g per side elastics was used full time and changed a minimum of twice per day. The elastic force was increased to 170 g. At 4 months, 230 g of elastics was used and continued until the end of the treatment.

### RME–FM treatment protocol

**Fig. 3:**
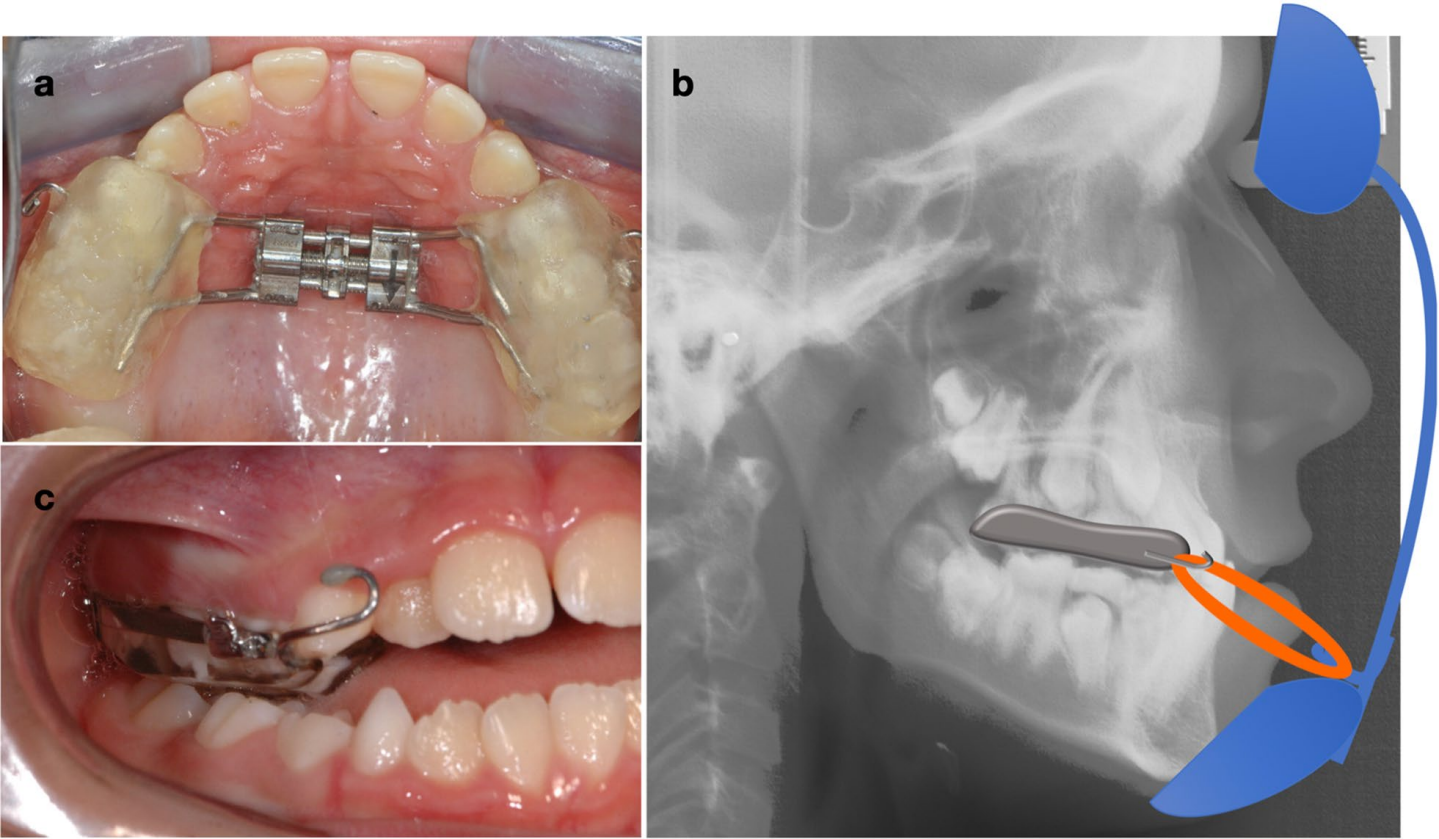
**A** Bonded Hyrax appliance with acrylic bite blocks; **B** Face mask hooks emerge near the maxillary canine for elastic application; **C** Face mask elastic force vector at approximately 30° down from the occlusal plane

The RME–FM group were treated with a bonded splint-type expansion appliance with hooks emerging near the maxillary canine area for the application of elastics to face mask (Fig. 3). The appliance resembled that previously published by Baccetti et al. [2], which used a Hyrax-type expansion screw (Dentaurum GmbH and Co. KG, Inspringen, Germany). Patients were instructed to turn the expansion mechanism twice a day until the desired expansion was reached. The face mask was adjusted so that the elastic force vector was angled at 30° down from the occlusal plane. Patients were then asked to wear the face mask for 14–16 h every day with an elastic force of 400 g/side.

### Cephalometric analysis

**Fig. 4:**
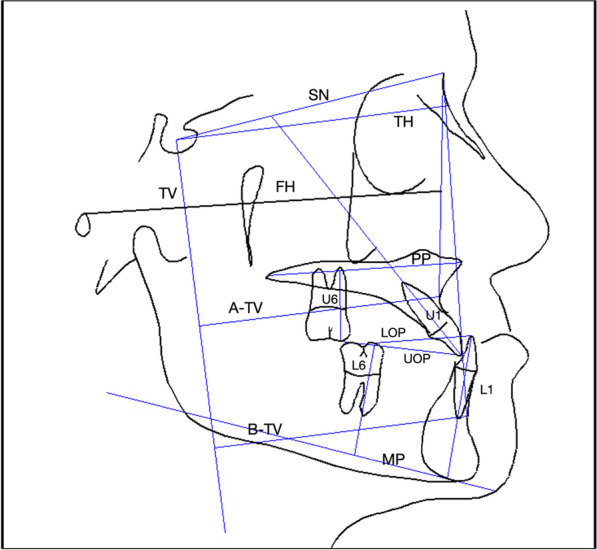
Cephalometric measurements and reference lines. *SN* Sella-Nasion line. *TH* true horizontal line 7° from SN. *TV* true vertical line 90° from TH through Sella. *A-TV* perpendicular distance from A point to TV, *B-TV* perpendicular distance from B-Point to TV, *FH* Frankfort horizontal line, *PP* palatal plane through ANS-PNS, *MP* mandibular plane, *UOP* upper occlusal plane: maxillary incisal tip to mesiobuccal cusp of first molar, *LOP* lower occlusal plane: mandibular incisor tip to mesiobuccal cusp of mandibular first molar. *U1* long axis of the most labial upper incisor, *L1* long axis of the most labial mandibular incisor, *U6* maxillary first molar long axis: mesiobuccal cusp to mesiobuccal root tip, *L6* mandibular first molar long axis: mesiobuccal cusp to mesial root tip

All cephalograms were digitised and traced by the same examiner (NET) using OrthoTrac imaging V11.7.0.32 (Carestream Dental, Atlanta, GA, USA). The cephalometric variables used in the analysis are illustrated in Fig. 4. Blinding was not possible for the end-of-treatment radiographs as skeletal anchors were in situ. The error of measurements was done on randomly selected lateral cephalograms of 11 patients, which were chosen using a random number generator. The intraclass correlation coefficients (ICC) showed excellent reliability, with values ranging between 0.981 and 0.999, except the L1-MP for which inter-rater reliability was still high, at 0.868 (Table 2).

**Table 2:** Error measurements

	ICC	%95 CI		Dahlberg's d	
		Lower	Upper	d	%
SNA	0.999	0.995	1.000	0.280	0.359
SNB	0.997	0.988	0.999	0.349	0.437
ANB	0.997	0.988	0.999	0.224	− 11.713
Wits	0.987	0.952	0.996	0.459	− 7.620
A-TV	0.984	0.937	0.996	0.748	1.288
B-TV	0.993	0.975	0.998	0.824	1.424
PP-MP	0.991	0.967	0.998	1.010	4.133
SN-MP	0.987	0.952	0.997	1.240	3.670
AR-Go-Me	0.984	0.945	0.996	1.602	1.195
UOP-PP	0.981	0.932	0.995	1.153	10.769
LOP-MP	0.946	0.804	0.985	1.532	7.307
U1-SN	0.994	0.980	0.999	1.005	0.962
U1-PP	0.995	0.983	0.999	0.996	0.876
U6-PP	0.981	0.934	0.995	1.286	1.604
L1-MP	0.868	0.537	0.964	3.578	4.200
L6-MP	0.990	0.963	0.997	1.311	1.671
Overjet	0.993	0.974	0.998	0.241	− 10.742
Overbite	0.998	0.992	0.999	0.235	15.699

The sample size was calculated using G*Power software (G*Power Version 3.1.9.6, Franz Faul, University of Kiel, Germany) based on previous data [20] and using a two-tailed *t* test and *α* = 0.05, power = 0.80, a sample size of 18 patients for each group was needed.

### Statistical analysis

IBM SPSS Statistics software (version 23.0. Armonk, NY: IBM Corp.) was used to analyse the data. Means and standard deviations are presented for all variables. Normality and homogeneity of variance of the data were assessed using Shapiro–Wilk's test and Levene's test for equality of variances, respectively. Differences between two timepoints within groups were tested for significance using a paired-samples *t* test. Differences between groups were tested for significance using an independent samples *t* test. The statistical significance level of *p* < 0.002 was chosen following the Bonferroni correction.

## Results

**Fig. 5:**
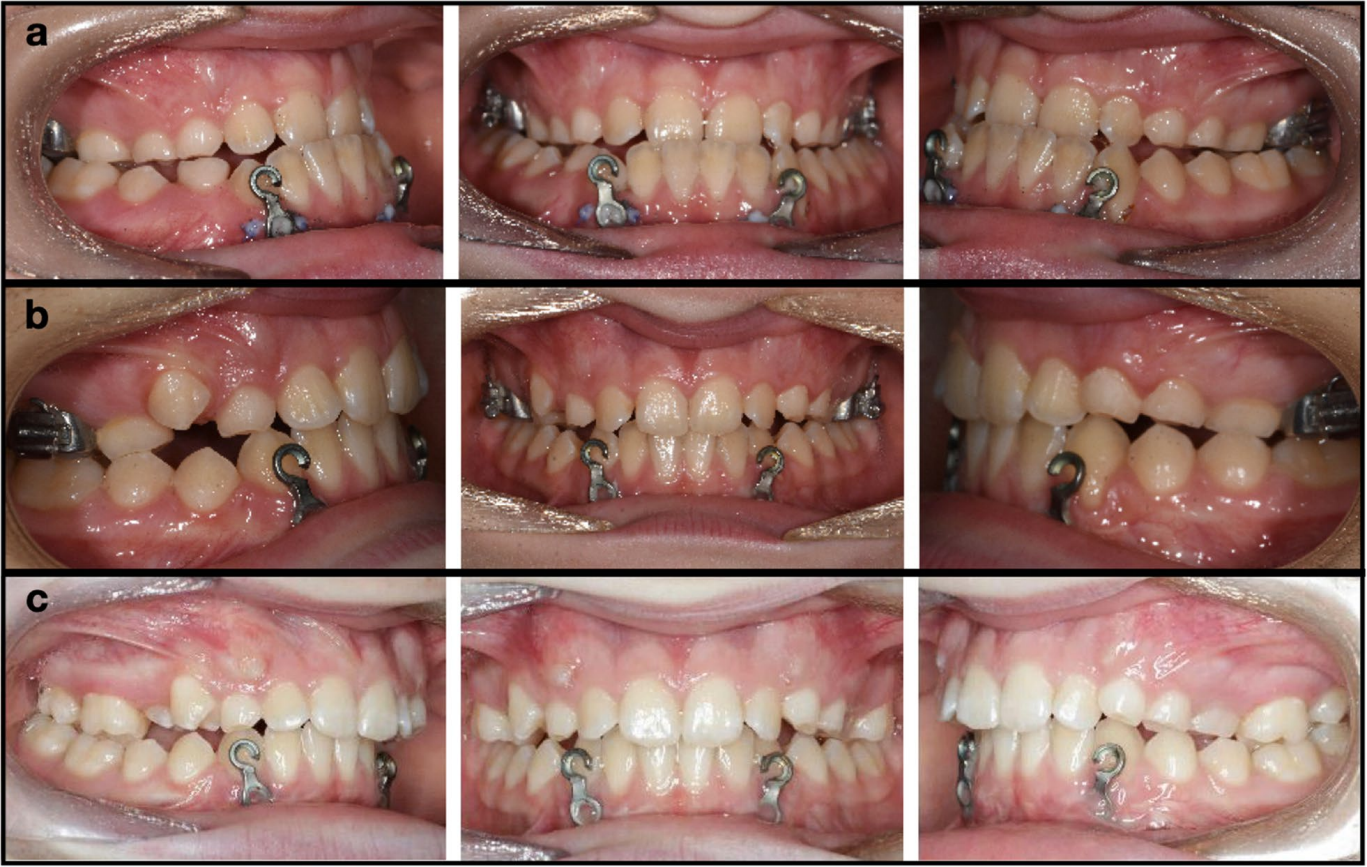
Progression of the case from the HE-MP group from start to finish. **A** Start of elastic wear after conversion of the miniplates into a hooks; **B** 7 months’ progress with positive overjet developing; **C** Treatment finished at 14 months with a positive overjet and slightly overcorrected molar relationship

**Fig. 6:**
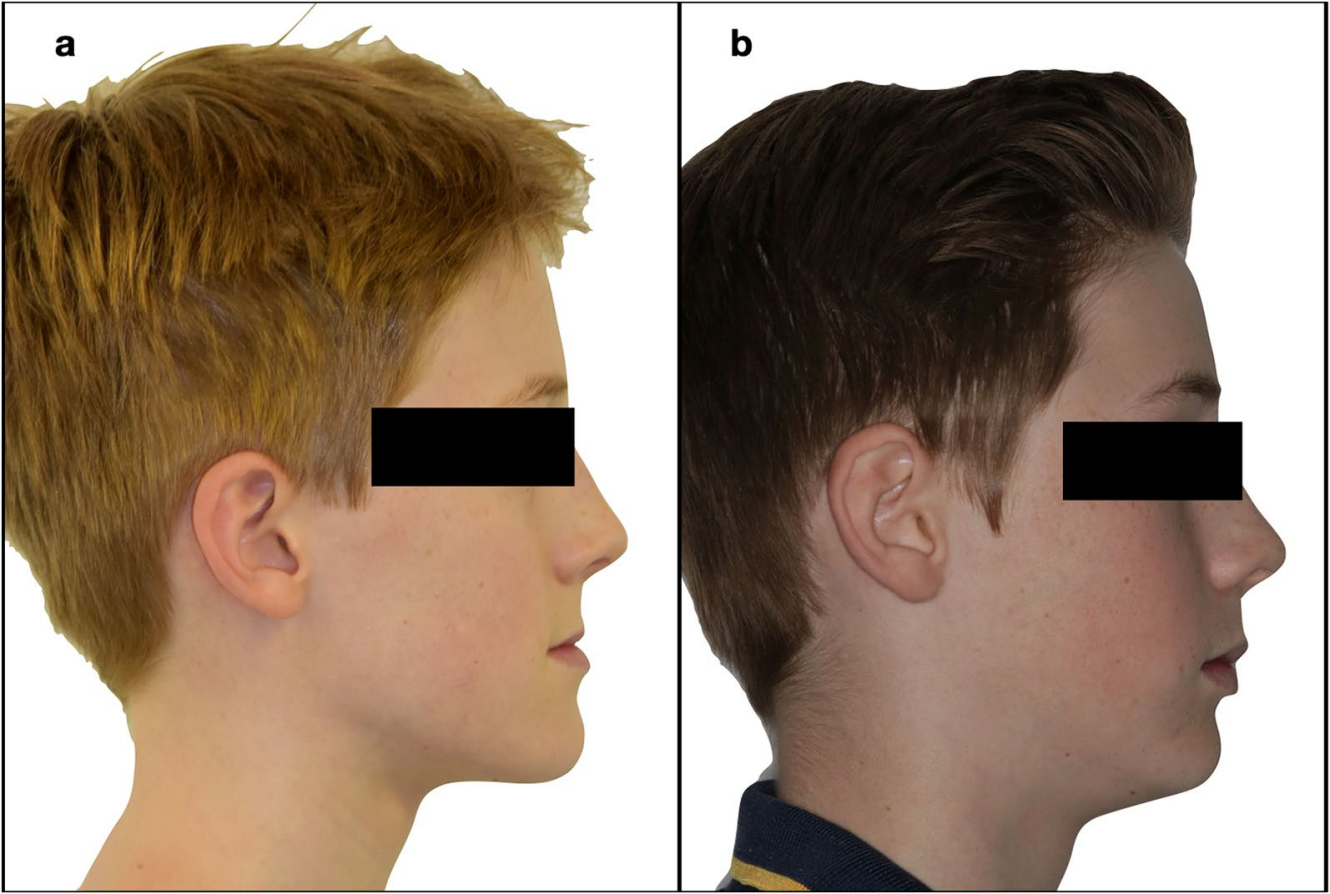
Before (**a**) and after (**b**) profile comparison. A significant increase in facial convexity and a reduction in chin prominence

The initial analysis of the skeletal and dental characteristics of the two groups before treatment (T1) showed no statistically significant differences between the two groups (Table 3), except for some borderline differences in (Ar–Go–Me) as well as for the occlusal plane to the mandibular plane angle (LOP-MP). The average treatment time was approximately 15.5 months (SD = 2.8) for the HE-MP group and 11.85 months (SD = 3.41) for the RME–FM group. Intraoral and extraoral images of a patient treated with HH-MP are displayed in Figs. 5 and 6.

**Table 3:** Descriptive statistics and baseline comparisons T1

	HH-MP		RME–FM		*p*
	Mean	SD	Mean	SD	
SNA	79.27	4.05	78.36	3.33	0.469
SNB	81.06	4.15	80.09	3.38	0.449
ANB	− 1.81	2.05	− 1.74	1.59	0.921
Wits	− 6.34	1.63	− 6.30	2.33	0.954
A-TV	57.73	4.62	58.68	3.87	0.51
B-TV	58.27	7.99	58.54	6.07	0.909
PP-MP	24.58	5.63	24.99	4.75	0.818
SN-MP	31.88	5.42	33.92	5.00	0.25
AR-Go-Me	132.06	6.40	136.50	4.78	0.024
UOP-PP	10.58	4.19	11.53	4.84	0.533
LOP-MP	17.89	3.81	20.65	4.05	0.043
U1-PP	115.34	8.15	112.71	5.87	0.274
U6-PP	79.27	4.60	81.19	3.52	0.169
L1-MP	88.59	6.85	85.96	6.52	0.245
L6-MP	78.83	7.08	77.22	5.73	0.457
Overjet	− 1.67	1.26	− 2.23	1.65	0.259
Overbite	0.75	1.74	1.67	2.53	0.212

Analysis of the changes experienced in both groups from T0 to T1 indicated significant differences between the two groups in terms of treatment response (Table 4).

**Table 4:** Treatment changes and comparisons within and between groups

	HH-MP (T2–T1)					RME–FM (T2–T1)					Difference between groups for T2–T1				
	Mean	SD	%95 CI		*p*	Mean	SD	%95 CI		*P*	Mean Diff	SE	%95 CI		*p*
			Lower	Upper				Lower	Upper				Lower	Upper	
SNA	4.26	2.15	3.19	5.33	< 0.001	1.14	0.93	0.68	1.61	< 0.001	− 3.11	0.55	− 4.25	− 1.97	< 0.001
SNB	− 0.96	1.23	− 1.57	− 0.35	0.004	− 0.89	1.21	− 1.49	− 0.29	0.006	0.07	0.41	− 0.76	0.89	0.87
ANB	5.25	2.03	4.24	6.26	< 0.001	2.04	1.07	1.51	2.58	< 0.001	− 3.21	0.54	− 4.32	− 2.09	< 0.001
Wits	6.03	3.13	4.48	7.59	< 0.001	2.94	1.75	2.07	3.81	< 0.001	− 3.09	0.84	− 4.81	− 1.38	< 0.001
A-TV	4.06	3.15	2.49	5.62	< 0.001	1.69	1.06	1.17	2.22	< 0.001	− 2.36	0.78	− 3.99	− 0.73	0.007
B-TV	− 1.43	2.56	− 2.71	− 0.16	0.03	− 0.92	1.86	− 1.84	0.01	0.052	0.52	0.75	− 1.00	2.03	0.494
PP-MP	1.40	2.78	− 0.03	2.83	0.055	1.52	2.74	0.16	2.88	0.031	0.12	0.93	− 1.78	2.02	0.897
SN-MP	0.48	1.87	− 0.45	1.41	0.292	1.13	1.90	0.18	2.07	0.022	0.65	0.63	− 0.62	1.92	0.307
AR-Go-Me	− 0.19	1.39	− 0.88	0.50	0.56	0.48	2.81	− 0.92	1.88	0.481	0.67	0.74	− 0.83	2.17	0.369
UOP-PP	− 0.12	3.03	− 1.63	1.39	0.866	− 1.44	3.01	− 2.94	0.05	0.057	− 1.32	1.01	− 3.37	0.72	0.198
LOP-MP	2.58	2.99	1.10	4.07	0.002	2.41	3.62	0.61	4.21	0.012	− 0.18	1.11	− 2.43	2.07	0.873
U1-PP	− 1.22	4.13	− 3.28	0.83	0.227	5.02	3.93	3.07	6.98	< 0.001	6.24	1.34	3.51	8.98	< 0.001
U6-PP	1.46	2.47	0.23	2.68	0.023	3.07	1.77	2.19	3.95	< 0.001	1.62	0.72	0.16	3.07	0.03
L1-MP	2.87	5.37	0.20	5.54	0.037	− 5.29	3.57	− 7.07	− 3.52	< 0.001	− 8.16	1.52	− 11.25	− 5.07	< 0.001
L6-MP	2.07	4.15	0.01	4.14	0.049	− 0.62	5.83	− 3.52	2.28	0.656	− 2.69	1.69	− 6.12	0.73	0.119
Overjet	4.12	1.48	3.38	4.86	< 0.001	5.19	1.86	4.27	6.12	< 0.001	1.07	0.56	− 0.07	2.21	0.064
Overbite	4.26	2.01	− 0.10	1.89	0.076	0.16	2.29	− 0.98	1.30	0.769	− 0.73	0.72	− 2.19	0.73	0.314

### Skeletal changes

Differences between the groups were significant for changes in SNA, ANB and Wits.

The antero-posterior assessment of the effect on the maxilla as assessed by the SNA angle and A-TV indicated a significantly greater skeletal advancement of the maxilla in the HE-MP group. There was an increase in SNA of 4.26° (SD = 2.15) and a 4.1 mm (SD = 3.1) increase in the A-TV measurement, as opposed to 1.14° (SD = 0.93) and 1.69 mm (SD = 1.06) in the RME–FM group (*p* < 0.001).

The effect on the mandible was similar in both groups, with the HE-MP group showing a reduction of 0.96° (SD = 1.23) in the SNB angle and 1.43 mm (SD = 2.56) in the B-TV, while the RME–FM group displayed a reduction of 0.89° (SD = 1.21) in the SNB angle and 0.92 mm (SD = 1.86) in the B-TV. The overall skeletal change was significantly greater in the HE-MP group, with an increase in the ANB angle of 5.25° (SD = 2.03) and an increase in the Wits appraisal of 6.03 mm (SD = 3.13). This was in comparison with increases of 2.04° (SD = 1.07) in the ANB angle and 2.94 mm (SD = 1.75) in the Wits appraisal in the RME–FM group (*p* < 0.001). In the vertical dimension there was slightly more increase in the vertical parameters for the RME–FM group, with a 1.13° (SD = 1.9) increase in the SN-MP angle and non-significant 0.48° (SD = 1.87) increase in the HE-MP group.

### Dental changes

The dental changes were significantly different for upper and lower incisor inclination changes and were in opposite directions. In the RME–FM group, there was an increase in U1-PP of 5.02° (SD = 3.93) (*p* < 0.001). However, there was retroclination of the upper incisor in the HH-MP group, though this was not significant. The maxillary molars (U6-PP) tipped mesially by 3.07° (SD = 1.77) in the RME–FM group and by 1.46° (SD = 2.47) in the HE-MP group. There was some counterclockwise rotation of the maxillary occlusal plane in the RME–FM group (− 1.44°; SD = 3.01) while there was no change in the HE-MP group. This, however, was not statistically significant. The mandibular incisor changes were significantly different between the two groups. The mandibular incisors (L1-MP) retroclined by 5.29° (SD = 3.57) in the RME–FM group, while they advanced slightly in the HE-MP group by 2.87° (SD = 5.37; *p* < 0.001).

### Stability of the miniscrews and miniplates

Only one palatal miniscrew (2.6%) failed in this study. The failure was not discovered until the completion of treatment, when the appliance was removed and the Beneplate retainer (PSM Medical Solutions, Gunningen, Germany) was to be placed. A new miniscrew was placed for retention. In five patients, the fixation screw (PSM Medical Solutions, Gunningen, Germany) fell out and had to be replaced; however, the Hyrax rings remained in place over the miniscrew.

Complications were experienced with 20% of miniplates, most of which were minor. Only one of the miniplates became loose during treatment. In one patient, gingival overgrowth around one miniplate had to be removed using a soft tissue laser and four patients experienced discomfort around the miniplates, mostly from gingival irritation. This, however, did not interfere with their ability to wear the elastics. In the patient shown in Fig. 5a–c, the initial photographs show (Fig. 5a) mild gingival recession following placement of the miniplates on the lower left canine, which healed over time (Fig. 5c); however, this should be examined in patients having MP.

## Discussion

The current study compared the skeletal and dental effects of two protocols in the correction of Class III malocclusion. The approaches differed significantly in the mode of force application used for maxillary protraction. The first approach (RME–FM) used a tooth-borne appliance with an extraoral face mask for part-time force application (14–16 h/day) while the HE-MP protocol relied on skeletal anchorage and the intraoral application of full-time elastic traction.

The skeletal changes shown in the RME–FM group in this study were similar to those reported by other studies using face mask therapy, with a 1.14° increase in the SNA angle, a 0.89° reduction in the SNB angle, an overall skeletal change of 2° in the ANB angle (Table 4). Other studies on the tooth-borne RME face mask have shown changes between 0.7° [21] and 1.8° for SNA [22–25]. The mandibular and overall skeletal changes as well as the significant dental changes observed in this study are also similar to others [2, 22–27].

The maxillary advancement was significantly higher in the HE-MP group, with more than threefold the increase in SNA angle than that observed in the RME–FM group. This was also reflected by the fact that the linear measurement in the HE-MP group displayed more than twice the advancement at A point that was observed in the RME–FM group. The effect on the mandible was similar in both groups, with a reduction in the SNB of approximately 1°. In addition, there was no significant change in the mandibular plane angle in the HE-MP group, while there was an increase in the mandibular plane angle in the RME–FM group. The greater skeletal changes seen in the HE-MP group can be attributed to the use of skeletal anchorage. A similar skeletal response was reported using the Hybrid Hyrax with a Mentoplate [13, 14] as well as with a face mask [23]. Similar differences between skeletal anchorage and traditional tooth-borne expansion and face mask were also found by Cevidanes et al. [6] when comparing the BAMP protocol to the face mask with maxillary expansion. They reported a 5.9 mm improvement in the Wits measurement in the BAMP group as opposed to only 3.6 mm with RME–FM and, similar to the HE-MP in this study, the majority of the skeletal change was due to maxillary protraction with minimal vertical change. The reduced vertical side effect in the HH-MP is useful in patients with increased face heights.

### Differences in dental effects

The use of skeletal anchorage significantly reduced the dental side effects in the maxillary dentition, with mild and non-significant uprighting of the upper incisors reported in the HE-MP group. These findings are consistent with those of other studies which have used hybrid expanders, where the use of palatal miniscrews to support expansion and protraction eliminated the maxillary dental side effects [14, 23]. It should be noted that the expansion protocols in the two groups were different. In the HH-MP group, since the maxillary expander was supported with palatal TADs, a slower rate of expansion was chosen, since the load of the appliance is mostly on the skeletal anchors, anchorage loss from dental movements is not expected [11]. Whereas in the RME–FM group, since the appliance is tooth borne, a rapid expansion protocol is utilised to be able to build up the forces to be high enough for sutural disarticulation, before dental movements take place. Nevertheless, there was a small amount of maxillary molar tipping observed with the HE-MP, which was similar to observations in other studies [14, 23]. This maxillary mesial molar tipping can be attributed to some wire bending and flexure of the appliance.

As in previous studies [2, 22–27], the mandibular incisors retroclined with the RME–FM. On the other hand, the mandibular incisors advanced slightly (on average) with the HE-MP. The standard deviation, however, shows that the response varied greatly between patients (Table 4). This variability was also seen in other studies [6, 14]. Willmann et al. [14] for example found that, on average, there was no change in the mandibular incisor inclination with Hybrid Hyrax-Mentoplate treatment, while Cevidanes et al. [6] reported a slight advancement of the lower incisors, which was similar to results in this study. This seems to be a finding that is unique to the use of skeletal anchorage plates in the mandible and may be attributed to two causes. Firstly, when Class III elastics are attached to the anchorage plates in the presence of an anterior crossbite, there is no direct force transmission to the lower incisors from the elastics. At the same time, the upper incisors are moving forwards as part of the downward and forward movement of the maxilla, and they may in turn indirectly push the lower incisors forward. Secondly, once the crossbite or edge-to-edge relationship is corrected and there is a positive overjet, there is a change in the tongue position, where it can now freely put pressure on the lingual surface of the lower incisors and move them to the newly established neutral zone between the lips and tongue [6].

The overjet reduction with the HE-MP was slightly less (1.07 mm; SD = 0.56) than what was seen with the RME–FM, despite the skeletal correction being greater in the HE-MP group. It was also noted that the treatment was, on average, 3 months longer with the HE-MP. This is likely due to the greater dental compensation associated with the RME–FM, which is achieved through upper incisor proclination and lower incisor retroclination, and which would lead to a faster development of a positive overjet. On the other hand, in the absence of dental compensation and even some mandibular dental decompensation, and with the correction almost exclusively stemming from skeletal changes, the overjet correction may take longer and show a smaller increase overall with the HE-MP. Similar results have also been shown with the BAMP protocol, where treatment has been recorded at an average of 2 months longer than with the face mask, with a smaller total correction in overjet [6]. It may be argued that for the long-term stability of the treatment result, this is a positive finding, as Class III patients tend to resume the original growth pattern when treatment is completed [22]. This lack of dental compensation may allow some room for future dental camouflage, should there be some relapse.

### Advantages of using HH-MP

Willman et al. found that when the Hybrid Hyrax-Mentoplate protocol was compared with the Hybrid Hyrax-face mask protocol, the results were very similar, except for more backward rotation of the mandible when the face mask was used. The face mask, however, has the significant limitation of reduced patient acceptance due to the obtrusive extraoral nature of the appliance [14].

Furthermore, even though the HE-MP protocol shares similar skeletal and dental effects with the BAMP protocol [6], there are several differences between the two. Firstly, there are four fewer surgical procedures involved with the HE-MP protocol, as the elimination of the zygomatic plates for maxillary anchorage using the hybrid expander halves the number of flap procedures. This also reduces the chances of miniplate failure, which has been reported to be six times higher in the maxilla than in the mandible [24, 25]. The higher failure rate of the maxillary zygomatic miniplates may be due to the difficulty in placing them in younger patients, due to reduced bone density [24–27]. On the other hand, the use of palatal miniscrews in the anterior palate to support the hybrid expander for the maxillary anchorage ensures the miniscrews are in an area of good bone quality [28, 29] where the success rate is high (more than 96%) and predictable, even in young patients [30]. Secondly, treatment with the HE-MP protocol can start earlier than with the BAMP protocol, which requires the mandibular canines to erupt prior to the placement of the miniplate. It is well documented that the maxilla is more responsive to protraction in younger patients, particularly those younger than 10 years old [31, 32]. The use of L-plates allows the miniplates to be placed before the eruption of the mandibular canines, thus allowing treatment in younger patients, much like what was reported with the use of Mentoplates [13]. Lastly, the HE-MP allows the incorporation of bone-borne maxillary expansion, which allows the concomitant management of any transverse maxillary deficiency (often present in maxillary hypoplasia [7] during Class III correction.

L-plates offer an advantage over the Mentoplate as well, since the right and left plates are independent of each other, this allows the surgeon more freedom to vary the position of the plates and find the best cortical bone. Furthermore, the use of traditional trauma plates, as opposed to proprietary plates such as the Mentoplates (PSM Medical Solutions, Gunningen, Germany) or the Bollard plates (Bollard; Tita-Link, Brussels, Belgium), may make the protocol more accessible to patients and potentially reduces the cost, as most surgical theatres will be equipped with traditional orthognathic trauma plates.

### Limitations

It is important to mention that the results of this study are limited to a short-term evaluation after treatment in two different centres. Long-term evaluation will be required to assess the stability of this treatment once the patients have completed postpubertal growth. It has been shown that face mask therapy is stable in 75–80% of cases in the long term [4, 21]. It remains to be seen if the greater skeletal response in the active treatment phase with this skeletal anchorage protocol results in better long-term stability.

The starting forms of the two groups were similar in all parameters except for Ar-Go-Me and LOP-MP angles. Whether or not this affected the treatment outcomes is unclear; however, an increased gonial angle is one of the predictors of failure in the long term for Class III patients and this should be investigated [33]. There was 1 patient in the HH-MP group, in whom compliance was poorer compared to others. However, since compliance of patients was not assessed for any of the patients, this patient’s data were still included in the results. It should again be noted that, in HH-MP patients, the dental effects are the opposite to RME–FM patients, which makes obtaining a positive overjet much more difficult.

It was also not possible to blind the T2 radiographs as images were collected with the skeletal anchors still in situ. Furthermore, the two groups were treated in different centres, so it can be argued that the outcomes may have differed; however, both treatment regimens followed universally used and accepted protocols. Another limitation of our study was the retrospective nature of the study; however, all patients fulfilling the inclusion criteria at the time of data collection were included.

## Conclusion

The results of this study indicate that, in the short term, the HE-MP approach produces a greater skeletal correction in Class III malocclusion in growing patients, with reduced dental side effects when compared to traditional tooth-borne RME–FM. Further studies with prospective designs as well as follow-up of the current patients are required into the long-term stability of these skeletal corrections.

## Data Availability

All data generated or analyzed during this study are included in this published article.
